# The effectiveness of peer mentoring in promoting a positive transition to higher education for first-year undergraduate students: a mixed methods systematic review protocol

**DOI:** 10.1186/s13643-016-0245-1

**Published:** 2016-04-22

**Authors:** Jean Carragher, Jennifer McGaughey

**Affiliations:** School of Health and Science, Dundalk Institute of Technology, Dublin Road, Dundalk, Co. Louth Ireland; School of Nursing & Midwifery, Medical Biology Centre, Queen’s University Belfast, 97 Lisburn Road, Belfast, Northern Ireland

**Keywords:** Educators, Higher education, Nursing, Midwifery, Mixed methods, Peer mentoring, Peer support, Transition

## Abstract

**Background:**

The global transfer of nursing and midwifery education to higher education institutes has led to student nurses and midwives experiencing challenges previously faced by traditional third-level students, including isolation, loneliness, financial difficulties and academic pressure. These challenges can contribute to increased stress and anxiety levels which may be detrimental to the successful transition to higher education, thus leading to an increase in attrition rates. Peer mentoring as an intervention has been suggested to be effective in supporting students in the transition to third-level education through enhancing a sense of belongingness and improving student satisfaction, engagement and retention rates. This proposed systematic review aims to determine the effectiveness of peer mentoring in enhancing levels of student engagement, sense of belonging and overall satisfaction of first-year undergraduate students following transition into higher education.

**Methods:**

MEDLINE, Web of Knowledge, ProQuest, Embase, CINAHL, ERIC, PsycINFO and CENTRAL databases will be searched for qualitative, quantitative and mixed methods studies on the implementation of peer assessment strategies in higher education institutes (HEIs) or universities for full-time, first-year adult students (>17 years). Included studies will be limited to the English language. The quality of included studies will be assessed using a validated Mixed Methods Appraisal Tool (MMAT). The findings will be presented as a narrative synthesis or meta-analysis as appropriate following sequential explanatory synthesis.

**Discussion:**

The review will provide clear, non-biased evidence-based guidance to all third-level educators on the effectiveness of peer-mentoring programmes for first-year undergraduates. The review is necessary to help establish which type of peer mentoring is most effective. The evidence from qualitative and quantitative studies drawn from the international literature will be utilised to illustrate the best way to implement and evaluate peer mentoring as an effective intervention and will be useful in guiding future research and practice in this area. These findings may be applied internationally across all disciplines.

**Electronic supplementary material:**

The online version of this article (doi:10.1186/s13643-016-0245-1) contains supplementary material, which is available to authorized users.

## Background

Nursing and midwifery education programmes worldwide have experienced significant change over the last few decades with the majority of pre-registration education programmes now delivered at third-level institutions in partnership with clinical sites [1–3]. This has led to a shift from traditional apprentice-style training to student nurses and midwives being perceived in the same way as any third-level student rather than as a trainee on the wards. Whilst there are many advantages to this style of education, there are also many challenges to face. Globally, student nurses and midwives are now struggling with the same problems faced by traditional third-level students [4]. These include financial difficulties, academic pressure and the sometimes stressful transition to higher education which can lead to high attrition rates, particularly in the health science professions [5–9].

Although the transition to higher education is seen in the main as a positive and exciting experience for first-year undergraduate students [10], it can also be a very challenging and stressful time [11–13]. For many, it is the first time to move away from home so students must adapt to new environments, make new acquaintances and friends and learn to survive independently, whilst at the same time coping with the academic demands of third-level education [10, 13, 14]. A negative transitional period can result in feelings of not ‘belonging’ which can eventually lead to debilitating symptoms of psychological distress [15]. This may in turn contribute to higher rates of student attrition [16, 17].

Much of the research in this area both nationally and internationally has focused on promoting retention and preventing attrition of students [18, 19]. However, the factors that predict success in higher education are varied and difficult to identify [18, 20]. Some of the research studies seek to explore how personality attributes may impact on a successful transition to higher education; for example, attachment styles [10], learner identities [21], self-confidence [22], high self-efficacy beliefs and motivation [23, 24] or traits of ‘agreeableness and conscientiousness’ [25] which were specific to nursing (p. 77). Other studies have explored wider issues in relation to this issue, for example, support from family and friends, financial restrictions, gender, age, and prior educational attainment [14, 18, 26].

A recent study for the Higher Education Authority in Ireland explored student progression in higher education [27]. This research took account of gender, social class background and educational attainment of students in the analysis of completion rates. One of the strongest findings to emerge was the important co-relation between successful completion and prior educational success in key subjects such as English and Maths. However, the authors are keen to stress the importance of the student experience as a crucial component in promoting successful transition to and progression within the higher education system. A more recently published Irish study found that students transitioning into higher education reported many challenges and these were reported to have a negative and potentially long-term impact on academic performance [28]. This literature demonstrates the multiplicity of factors involved in relation to successful transition to higher education and highlights the importance of the first-year experience within this.

Prioritising the first-year experience in an attempt to promote a positive transition to higher education has been identified as crucial both nationally and internationally [29–31]. In the Republic of Ireland, the publication of the National Strategy for Higher Education to 2030 [32] emphasised the value of support during the first year. This has led to the development of many interventions in the higher education sector focusing on the first-year experience, in particular the initial transition stage. These support interventions aim to enhance staff-student relationships and promote student engagement [33, 34]. Such strategies include induction days/weeks; ice-breaking activities; seminars, student-centred learning and teaching strategies; and peer-mentoring schemes to name but a few [18]. One of the key strategies reported in the literature as being effective in enhancing the transition to higher education is that of peer mentoring, and this intervention is worthy of further exploration [28, 35].

The concept of mentoring has many confusing definitions and typologies [36]. For the purpose of this review, mentoring will be considered as a relationship ‘in which an individual with more expertise provides knowledge and information to a less experienced individual’ [37 p. 351]. Peer-mentoring programmes appear to play an important role in enhancing the student experience overall and supporting the transition to higher education [38]. A literature review by Clark and Andrews [36] explored the wider context and background around peer mentoring. This review was followed up by a 3-year research study into peer mentoring carried out at six higher education institutes (HEIs), five of which were in the UK and one of which was in Norway [35]. The findings from this research demonstrate clear evidence that peer mentoring helps in supporting the transition into higher education. In the Irish context, a recently published study found that students rated peer-mentoring schemes very highly in supporting their transition to higher education [28]. These positive findings are also supported by an action research study undertaken in two Institutes of Technology in Ireland exploring the first-year experience of a peer-assisted learning programme [39].

Whilst these are very positive findings, it is important to assess whether this research is supported by other findings internationally. It is timely then to undertake a systematic review of the literature specifically on the effectiveness of peer mentoring in relation to the transition to higher education. There are many outcomes to consider in relation to peer mentoring and the successful transition to higher education. It is suggested that the most important outcomes are those from the students’ perspective including enhanced engagement, sense of belongingness and overall satisfaction [40, 41]. These outcomes are now seen as central to improving the transition to higher education with the overall aim of reducing attrition rates [42].

## Research question

What are the effects of peer mentoring on first-year undergraduate students’ psychosocial well-being, skills acquisition and psychological health following transition into higher education?

## Aim

The aim of this review is to determine the effectiveness of peer mentoring in enhancing levels of psychosocial well-being, skills acquisition and psychological health of first-year undergraduate students following transition into higher education.

## Objectives

1. To evaluate the effectiveness of peer mentoring in promoting a positive transition to higher level education for first-year undergraduate students
2. To compare the effectiveness of different peer-mentoring interventions in promoting a positive transition to higher level education for first-year undergraduate students
3. To explore the range of contexts in which this intervention is delivered to determine how this may affect mechanisms and outcomes within the studies [43]

## Methods

A mixed methods review which ‘concomitantly examines qualitative, quantitative and mixed methods primary studies’ [44 p. 530] will be undertaken. Incorporating diverse forms of evidence from different types of research within a review can help strengthen the findings and make them more applicable to policy and practice [45]. Mixed methods research strengthens research evidence as it provides more than one type of research design to answer the research question, enhances validity through triangulation of data, provides an opportunity to use qualitative evidence to support and substantiate quantitative findings, provides a richer understanding of phenomenon than evidence derived from one type of research design and generates a more robust conclusion [46]. Methods for informing the mixed methods review are still emerging; therefore, this protocol is informed by evidence from a wide range of sources [45, 47, 48] and will follow the transparent and scientific steps recommended for the systematic review process [49–52] in accordance to the Preferred Reporting Items for Systematic Reviews and Meta-Analyses Protocols (PRISMA-P) checklist (Additional file 1). The importance of adhering to these systematic processes in combining quantitative and qualitative data will help to reduce the risk of bias as well as enhance the quality and future contribution of this review [51, 53]. As there are no specific health-related outcomes, we did not register the protocol with PROSPERO.

## Inclusion and exclusion criteria

### Types of studies

Randomised and non-randomised studies that assess the effectiveness of peer mentoring as an intervention to support first-year undergraduate students on their transition to higher education/university will be included. Randomised controlled trials (RCTs), quasi-controlled trials, controlled before-and-after (CBA) studies, interrupted time series (ITS) studies, cohort studies, case-control studies and surveys will all be considered. The inclusion of qualitative studies in this review will help establish the important relationships between the intervention and context within the studies and will inform and enhance the overall findings [43, 54]. Qualitative methodologies will include case studies, phenomenology, grounded theory, ethnography and action research studies. Studies using a mixed methods design will also be considered for inclusion.

### Types of participants

Full-time, first-year students of all disciplines who enter a programme of higher education in HEIs or university and are over the age of 17 will be included. Students in primary- and secondary-level education will be excluded.

### Types of interventions

Studies will be included if they involve any type of peer-mentoring programme for first-year undergraduate students within a higher education institute or university. Peer-mentoring programmes include group or individual interventions and either face-to-face or interventions aided by technology (either telephone or online). Studies that compare any peer-mentoring intervention with a control group (usual support) or one type of peer-mentoring intervention versus another will also be included.

### Types of outcome measures

Outcome measures of peer support classified by Sartore [55] as psychosocial well-being, skills acquisition and psychological health will be reported. Psychosocial well-being factors specifically address how the student has adapted to the transition to third-level education and include outcome measures of ‘belongingness’ to the institution and discipline, overall student satisfaction, level of engagement, personal functioning/coping and their perceived social support. Skills acquisition outcomes include an increased confidence and skill in transition to HEI or placement and knowledge of support services/resources in HEI as well as an improvement in the end of semester/year academic performance. Levels of anxiety, depression, confidence and self-esteem [56] as factors of psychological health will be reported. All other outcomes reported in the original studies will also be considered.

The outcomes or end points reported may be affected by factors related to causal relationships between mechanisms and contexts, so these will be included in the review where appropriate [55] to explain why peer mentoring works (or not), for whom and in what circumstances [43, 54]. Mechanisms are determined by the actions undertaken by individuals in particular contexts based on their reasoning and the resources available to achieve a desired outcome [57], and Petticrew et al. [43] define context as being ‘the particular geographical, cultural, and social environment and the organisational and political systems in which an intervention or program takes place’ (p. 1233). These mechanisms and contexts may include any number of factors that influence the development and success of peer-mentoring programmes, for example, facilitators and barriers to uptake of peer-mentoring interventions and social, cultural, political or environmental issues. It will also be important to consider participants’ experiences of peer-mentoring interventions. It is envisaged that these factors will be primarily explored through qualitative data (possibly within mixed methods studies) [55].

## Search strategy

A search of MEDLINE (1946 to present), Web of Knowledge (1980 to present), ProQuest (1980 to present), Embase (1980 to present), CINAHL (1950 to present), ERIC (1995 to present), PsycINFO (1980 to present) and CENTRAL (The Cochrane Library, latest issue) will be undertaken to comprehensively search for studies on the implementation of peer assessment strategies in HEIs or universities. The search terms will include controlled vocabulary or Medical Subject Heading (MeSH) terms and synonyms, related words or free text words for peer-mentoring student and higher education as appropriate (Table 1).

**Table 1:** MEDLINE search terms

‘peer mentor$’.mp.	
‘peer support’.mp.	
(‘buddies’ or ‘buddy’s’)	
1 OR 2 OR 3	
students.mp. or Students/	
‘higher education students’.mp.	
‘university students’	
5 OR 6 OR 7	
4 AND 8	

The MEDLINE database will be searched first, and subsequent searches will be adapted from the MEDLINE strategy to ensure transparency and achieve a sensitive search [58]. To ensure a broad search is undertaken, the text words will be searched for across the entire paper rather than limiting to title, abstract and background. No methodological filter will be applied as all methodologies will be considered. English language articles only will be considered for ease of interpretation.

A search of grey literature will include contacting experts where appropriate and hand searching of particular journals with a focus/supplement on peer mentoring, for example, The All Ireland Journal of Teaching and Learning, Journal of Further and Higher Education, Journal of Nursing Education (JNE) and Nurse Education Today. Citation searching will also be undertaken to locate important studies [53] and a search of specific websites: Higher Education Authority (Ireland)/National Forum for Teaching and Learning (Ireland)/The National Forum for the Enhancement of Teaching and Learning in Higher Education (Ireland)/Higher Education Academy (UK) for government papers on education/conference papers over the past 10 years [52, 58].

## Data screening and extraction process

All titles retrieved from the initial search will be screened by one reviewer and irrelevant studies excluded. All studies which meet the eligibility criteria will be screened by two reviewers independently. Any conflict will be resolved via third party arbitration. A pre-determined study eligibility form and data collection tool will be used and consistently applied by two reviewers to reduce selection bias (see Additional file 2). A pilot of the eligibility criteria and data collection form will be undertaken on a sample of reports independently by two reviewers to assess validity and reliability [59]. An additional file provided shows this in more detail. The rationale for exclusion of studies will be documented, and a Preferred Reporting Items for Systematic Reviews and Meta-Analyses (PRISMA) flowchart will be utilised to illustrate the process by which studies were selected [60].

Data extraction will include the characteristics of the study in order to map the research in this area as a preliminary stage to synthesis. In addition, the findings or outcomes of each study will be recorded in preparation for analysis and synthesis [52]. It is proposed that the data extraction will use a process described by Sandelowski et al. [61] to ensure that the qualitative and quantitative data are transformed into statements that preserve the methodological context of findings when reporting mixed methods research synthesis. This approach ensures that text and content information regarding the sample, time, comparators, magnitude and significance and concepts of phenomena are integrated into useable statements or iterations of the findings.

## Quality assessment

Quality appraisal of the included studies will follow a three-stage process which includes filtering, technical appraisal and theoretical appraisal [52, 62, 63]. The filtering process assesses the relevance of the study to the review question (external validity) which is undertaken during data screening. Whilst all of the studies may fit the inclusion criteria, some will be less relevant than others due to aspects of the study design [52].

Technical appraisal will assess the appropriateness or soundness of the study design and methods in addressing the review question. This process will examine criteria related to the rigour of the data collection and analysis, sampling procedures, findings, reflexivity of researcher and adequacy of conclusions [62]. High-quality RCTs may be less relevant to answering the research question than other types of research studies, and therefore, the studies which are most appropriate to answer the research question rather than the hierarchy of evidence will be used to determine quality [64]. The rationale is to ensure applicability across contexts rather than focusing on one form of evidence [45]. Theoretical appraisal will assess how the study design has been operationalised in relation to the underpinning theoretical framework. This will provide judgement on the coherence and consistency between the methods, findings and interpretation that guide the research study [62].

As this review incorporates mixed methods studies, it is proposed to utilise an appraisal tool that incorporates criteria for appraising the quality of all the different study types [44, 65]. The Mixed Methods Appraisal Tool (MMAT) [66] will be used as it has established content validity, has been piloted across all methodologies and is applicable to this review [44, 66, 67]. All included studies will be independently assessed to determine the quality and relevance of each study by two reviewers independently. All studies appraised will be categorised using the descriptors of the scoring metric of the MMAT as follows: (*) one criterion met to (****) all criteria met. Any disagreements will be resolved by discussion or if required by a third party [64]. The MMAT will assess all quantitative studies including randomised controlled trials (RCTs), non-randomised and descriptive [44, 66, 67].

The MMAT will appraise all qualitative studies [44, 66, 67] in a systematic and transparent manner [62, 64] and retain the respect for the philosophical traditions of qualitative research [67, 68]. To ensure qualitative philosophical traditions are respected and prevent useful rich data being excluded from the review, at least one reviewer will have experience undertaking and appraising qualitative research [68, 69].

Mixed methods studies will be appraised using the MMAT which utilises specific criteria designed to elicit how well the qualitative and quantitative components of mixed methods studies are integrated [66].

## Data analysis

### Data analysis of quantitative studies

Meta-analysis of appropriate quantitative data will include calculating a summary statistic; for dichotomous data, this will be calculated as a risk ratio (RR) and 95 % confidence intervals (CIs). If any results are reported as odds ratios, they will be converted to risk ratios before interpretation [55, 70]. For continuous data, the summary statistic will be calculated as a difference between means.

Ordinal outcome data may be encountered in the form of scales. If appropriate, these scales will be checked to determine if they allow for modification to dichotomous data. Larger ordinal scales will be treated as continuous data [55, 70]. If count measures are found in this review, they will be considered as continuous data [55]. Meta-analysis will be undertaken for studies deemed sufficiently homogenous. Review Manager 5 software [71] will be used to present data using forest plots and to calculate the overall summary statistic. Heterogeneity will be calculated using *I*^2^ and standard chi-square test. Sensitivity analyses will be performed to examine the effects of excluding subgroups or risk of bias if indicated during the review process [70]. All findings will be tabulated into a ‘Summary of Findings’ table to provide information regarding the quality of evidence, magnitude of effect and data on important outcomes for a given comparison [72]. Where it is not appropriate to use meta-analysis, a narrative synthesis will be undertaken for heterogeneous quantitative studies [73–75] or when there is a wide variety of diverse studies to be included [76].

### Data synthesis of qualitative studies

A three-step narrative synthesis approach described by Popay et al. [77, 78] will be used: developing a preliminary synthesis, exploring relationships in the data and assessing the robustness of the synthesis product. A preliminary synthesis of the findings of the included studies will be undertaken during the data extraction stage. This will allow for an initial description of the results of included studies and will begin to explore how and why this intervention worked (or not) [61, 78]. In the preliminary synthesis, the reviewers will organise the results of the studies by identifying and describing textually, maintaining ‘text in context’ [61]. This will highlight the findings and correlations in and across the included studies maintaining context. Once patterns begin to emerge across study results, the reviewers will commence a rigorous synthesis exploring the relationship between the findings. These include exploring the context, mechanism and outcomes of the different studies to assess differences and similarities to examine heterogeneity. This will allow the reviewers to detect emerging patterns across the studies in relation to peer mentoring and determine which factors/processes impacted on effectiveness as a result of the synthesis [78]. This will allow the findings to be synthesised to develop a theory of how the programme works in what circumstances for whom [43].

Finally, the robustness of the synthesis produced will be assessed [76, 77, 79, 80]. Robustness will be assessed by evaluating the methodological quality of the primary studies included in the synthesis through the use of the MMAT. In addition, the reviewers will undertake a critical reflection on the synthesis process, examining the methodology used and exploring any limitations or biases. This will result in an overall assessment of the strength of the evidence available allowing conclusions to be drawn on the effectiveness of peer mentoring in relation to the differing contexts, mechanisms and outcomes as outlined in the review objectives.

### Synthesis of studies

The quantitative and qualitative data extracted will be synthesised and integrated. The synthesis of disparate evidence has been demonstrated to be a very challenging process [43, 48, 76–81]. As a result, a multilevel or parallel syntheses approach, recommended by Noyes [54] and utilised by Thomas et al. [82] and Noyes and Popay [83], respectively, will be undertaken as an alternative choice to the Bayesian method for integrating different methods in systematic reviews [45]. This three-stage process will include meta-analysis (if deemed appropriate), narrative synthesis of qualitative findings and synthesis combining both sets of data as illustrated by Thomas et al. [82]. This segregated framework for the synthesis of mixed methods studies as described by Sandelowski et al. [84] has been adopted by the Joanna Briggs Institute (JBI) guidelines [45]. Pluye and Hong [85] use the label “sequential explanatory synthesis” to describe this method. This approach has been used successfully to integrate quantitative and qualitative findings in many recent systematic reviews making it a pragmatic option for this review [86–89]. To ensure transparency, the synthesis process will be described and documented [52, 62].

## Discussion

This protocol outlines the process to be undertaken for a mixed methods review of peer mentoring. A mixed methods approach will allow a wider range of studies to be included to present a comprehensive understanding on the use of peer mentoring as an intervention for first-year undergraduate students. However, the synthesis of data from a range of diverse methodologies is complex and may lead to practical issues during synthesis. To address any practical problems, the synthesis of data will be reviewed independently by two independent reviewers and the process will follow JBI guidelines.

The findings of the review will be utilised to develop and implement an evidence-based research study on peer mentoring for midwifery students who experience similar transition issues to any other first-year undergraduate student entering higher education. The proposed Midwifery Peer Assisted Learning and Support (My PALS) Project will implement and evaluate a formal structured approach to peer mentoring and support in the undergraduate midwifery education programme of a selected HEI. It is anticipated that the research will help inform educators and other interested stakeholders across all disciplines on the benefits and challenges of introducing a peer-mentoring scheme for first-year undergraduate students. The project also has the potential to add to the existing body of knowledge around specific issues/concepts in nursing and midwifery education, for example, leadership, emotional intelligence, attrition, retention, belongingness, empowerment and compassionate care.

One of the limitations of this review is that the search strategy has been restricted to the English language. As a result, the generalisability of the review findings will be limited. Second, all studies which meet the inclusion criteria will be quality appraised and included in the review, i.e. no studies will be excluded on the basis of quality. The risk of bias and lack of rigour are primary concerns when undertaking a mixed methods review hence the necessity to demonstrate the steps followed in a clear and transparent manner throughout [45]. To ensure transparency and enhance rigour, a table clearly indicating the quality of all included studies will be developed. In addition, the data extraction form to be utilised across all studies may be impractical. As a result, the tool will be piloted and amendments will be made accordingly.

Systematic reviews provide a rigorous framework for combining research findings across several studies [90]. It is anticipated that this mixed methods review will give clear and non-biased guidance, based on sound evidence synthesis [91] to third-level educators across all disciplines about the effectiveness of peer mentoring as an intervention for first-year undergraduates. Literature from different methodologies will be utilised to illustrate the best way to implement and evaluate peer mentoring as an effective intervention and should also be able to guide future research in this area. The integration of different types of evidence in a systematic, transparent way should help shed new light on this intervention. This review should therefore be able to demonstrate not just if peer mentoring works ‘but how it works, in what population/subpopulations, and in what circumstances and contexts’ [43 p. 1231].

## Additional files

Additional file: 1 PRISMA-P (Preferred Reporting Items for Systematic review and Meta-Analysis Protocols) 2015 checklist. The file contains a list of recommended items to address in a systematic review protocol. (DOCX 15 kb) Additional file: 2 Study Selection, Data Collection and Quality Assessment Form. This is a pre-determined study eligibility form and data collection tool that will be used and consistently applied to reduce selection bias. (DOCX 1051 kb)

## Abbreviations

CBA: controlled before-and-after
CIs: confidence intervals
HEIs: higher education institutes
ITS: interrupted time series
JBI: Joanna Briggs Institute
MeSH: Medical Subject Heading
MMAT: Mixed Methods Appraisal Tool
My PALS: Midwifery Peer Assisted Learning and Support
PRISMA: Preferred Reporting Items for Systematic Reviews and Meta-Analyses
RCTs: randomised controlled trials
RR: risk ratio
