# Supplementary material for: The effectiveness of peer mentoring in promoting a positive transition to higher education for first-year undergraduate students: a mixed methods systematic review protocol
Source: Syst Rev. 2016 Apr 22;5:68. doi: 10.1186/s13643-016-0245-1 (PMC4840870; doi:10.1186/s13643-016-0245-1)

**ADDITIONAL FILE 2**

**Study Selection, Data Collection and Quality Assessment Form**

Name of author extracting data:

Date form completed:

**Study ID**

| **Title:** Family name of first author and year of publication + letter if more than one per year, eg. Smith 2001 b) |  |
| --- | --- |
| **Study ID** |  |
| **Are there other articles of same study?** (YES, NO, Unclear. If Yes, write Study IDs) |  |

**Study Eligibility**

|  | (please circle or state) | *Source (page no. in report)* |
| --- | --- | --- |
| **Type of study: Quantitative: (please circle)**  Randomized control trial (RCT)  Quasi-controlled trial  Controlled before-and-after (CBA) study Interrupted time series study (ITS)  Cohort study  Case control study  Other (specify) |  |  |
| **Type of study: Qualitative: (please circle)**  Case study  Phenomenological study  Grounded theory study  Ethnographic case study  Action research study  Other (specify) |  |  |
| **Type of study:**  Mixed Methods Research (MMR) (specify type) |  |  |
| **Participants**  Undergraduate students?  First year of studies at higher education/university?  Aged 17 or over? | **Yes, Unclear, No**  **Yes, Unclear, No**  **Yes, Unclear, No** |  |
| **Interventions: Types of Peer mentoring (Please circle)**  Face to face: group  Face to face: one to one  Telephone (text)  Telephone (call)  Online**:** group  Online: one to one  Other (please specify) |  |  |
| **Primary Outcomes (Psychosocial wellbeing): Did the study report any one of –**  1. Student engagement  2. Sense of belongingness  3. Student satisfaction  4. Any other related outcomes? (specify) | **Yes, Unclear, No**  **Yes, Unclear, No**  **Yes, Unclear, No**  **Yes, Unclear, No** |  |
| **Secondary outcomes (Skills acquisition):**  **Did the study report any one of –**   1. Increased confidence/skill 2. Improved knowledge of support services/resources in HEI 3. Improvement in academic performance. 4. Any other related outcomes? (specify) | **Yes, Unclear, No**  **Yes, Unclear, No**  **Yes, Unclear, No**  **Yes, Unclear, No** |  |
| **Secondary outcomes (Psychological health):** **Did the study report any one of –**   1. Anxiety/depression 2. Self-esteem. 3. Any other related outcomes? (specify) | **Yes, Unclear, No**  **Yes, Unclear, No**  **Yes, Unclear, No** |  |
| **Process/contextual outcomes: Did the study report any one of –**   1. Facilitators/barriers to uptake of peer mentoring 2. Social, cultural or environmental issues. 3. Participants experiences 4. Any other related outcomes? (specify) | **Yes, Unclear, No**  **Yes, Unclear, No**  **Yes, Unclear, No**  **Yes, Unclear, No** |  |
| **Conclusion:** If any of the above answers are NO do not continue: Document if study is to be included or excluded. If included continue to **Data Extraction Form**.  ** Included**  ** Excluded**  ** More information needed before inclusion decision (specify):** | | |

**Setting**

| **Country/area** |  |
| --- | --- |
| **Setting**  **(Type of HEI)** |  |
| **Discipline within HEI** |  |

**Participants (Student mentees)**

| **No of participants:**  % Male:  % Female |  |
| --- | --- |
| **Age (**mean/SD) |  |
| **Sex of participants**  (M/F numbers or %) |  |
| **Year of study** |  |
| **Race/ethnicity if recorded** |  |
| **Additional demographics:** |  |

**Participants (Student mentors)**

| **No of participants:**  % Male:  % Female |  |
| --- | --- |
| **Age (**mean/SD) |  |
| **Sex of participants**  (M/F numbers or %) |  |
| **Study discipline**  **And year** |  |
| **Race/ethnicity if recorded** |  |
| **Additional demographics:** |  |

**Additional notes:**

**Intervention**

| **Types of Peer mentoring** | **Description** |
| --- | --- |
|  |  |
|  |  |
|  |  |

**Outcomes: Continuous data**

| **Primary Outcomes**  **(specify continuous data below)** | **Unit of**  **measurement** | **Intervention Group** | | | **Control Group** | | |  | **95% CI** |
| --- | --- | --- | --- | --- | --- | --- | --- | --- | --- |
|  |  | **n** | **Mean**  **(SD)** | **Median (IQR)** | **n** | **Mean (SD)** | **Median (IQR)** | **p-value** |  |
|  |  |  |  |  |  |  |  |  |  |
|  |  |  |  |  |  |  |  |  |  |
|  |  |  |  |  |  |  |  |  |  |

| **Secondary Outcomes**  **(specify continuous data below)** | **Unit of**  **measurement** | **Intervention Group** | | | **Control Group** | | |  | **95% CI** |
| --- | --- | --- | --- | --- | --- | --- | --- | --- | --- |
|  |  | **n** | **Mean**  **(SD)** | **Median (IQR)** | **n** | **Mean (SD)** | **Median (IQR)** | **p-value** |  |
|  |  |  |  |  |  |  |  |  |  |
|  |  |  |  |  |  |  |  |  |  |
|  |  |  |  |  |  |  |  |  |  |

**Dichotomous data**

| **Primary Outcomes**  **(specify dichotomous data below)** | **Intervention Group**  **(n = )** | **Control Group**  **(n = )** | **p-value** | **Any further information** |
| --- | --- | --- | --- | --- |
|  |  |  |  |  |
|  |  |  |  |  |
|  |  |  |  |  |

| **Secondary Outcomes**  **(specify dichotomous data below)** | **Intervention Group**  **(n = )** | **Control Group**  **(n = )** | **p-value** | **Any further information** |
| --- | --- | --- | --- | --- |
|  |  |  |  |  |
|  |  |  |  |  |
|  |  |  |  |  |
|  |  |  |  |  |

Key conclusions of the study authors/other relevant information:

**Qualitative/quantitative descriptive data:**

Research aim, questions or objectives:

Theoretical framework:

Data collection methods:

Data collection tools and domains assessed:

Findings (For all qualitative reports and quantitative descriptive reports include a brief overview of results/themes).

Key conclusions of the study authors/other relevant information:


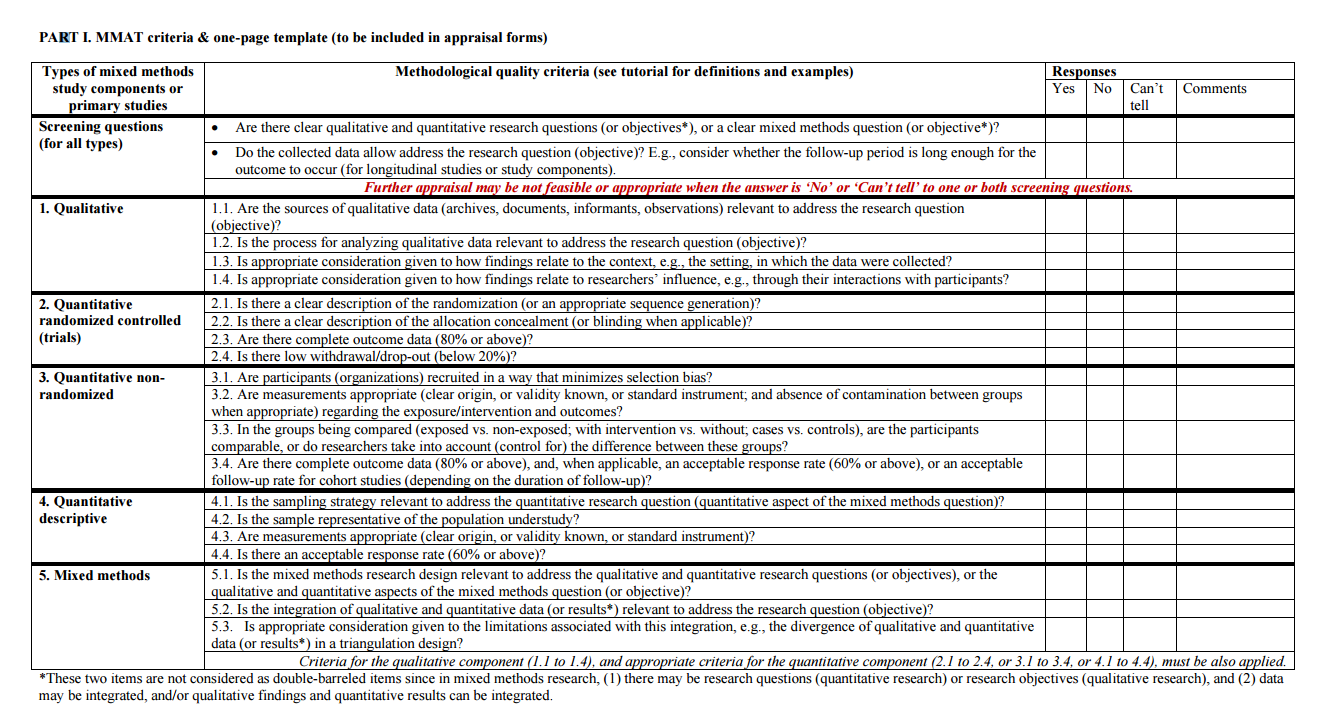


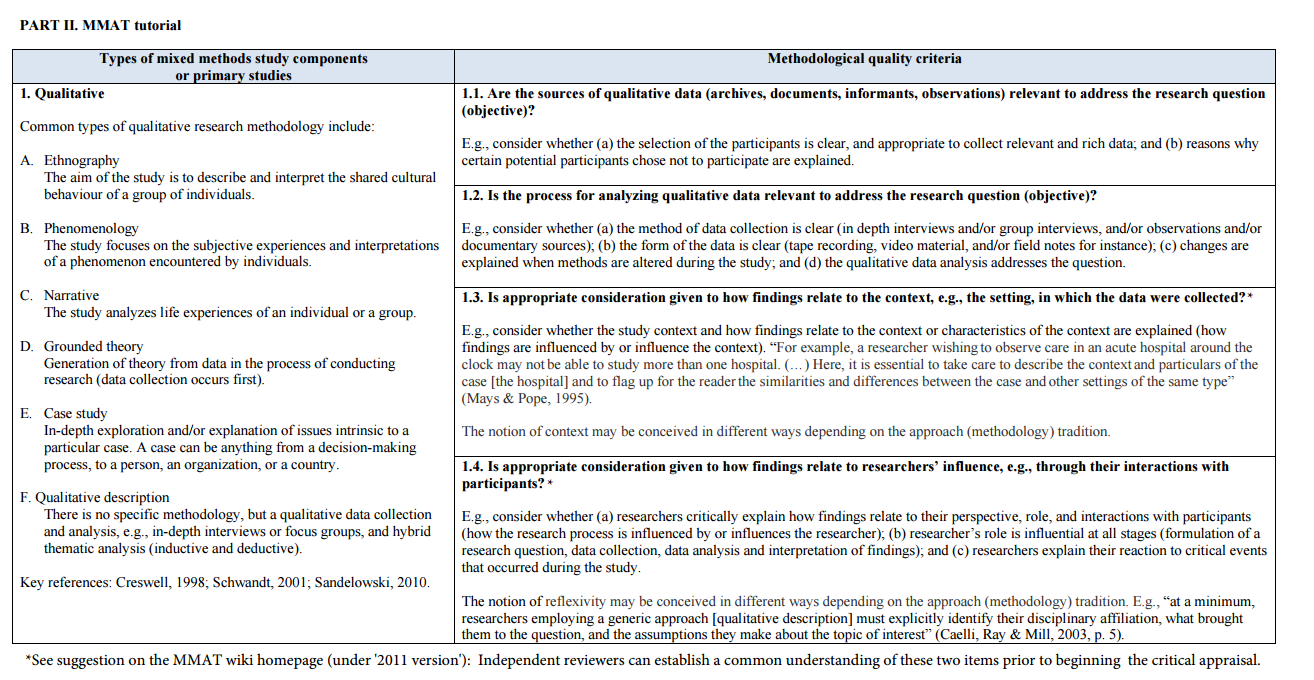


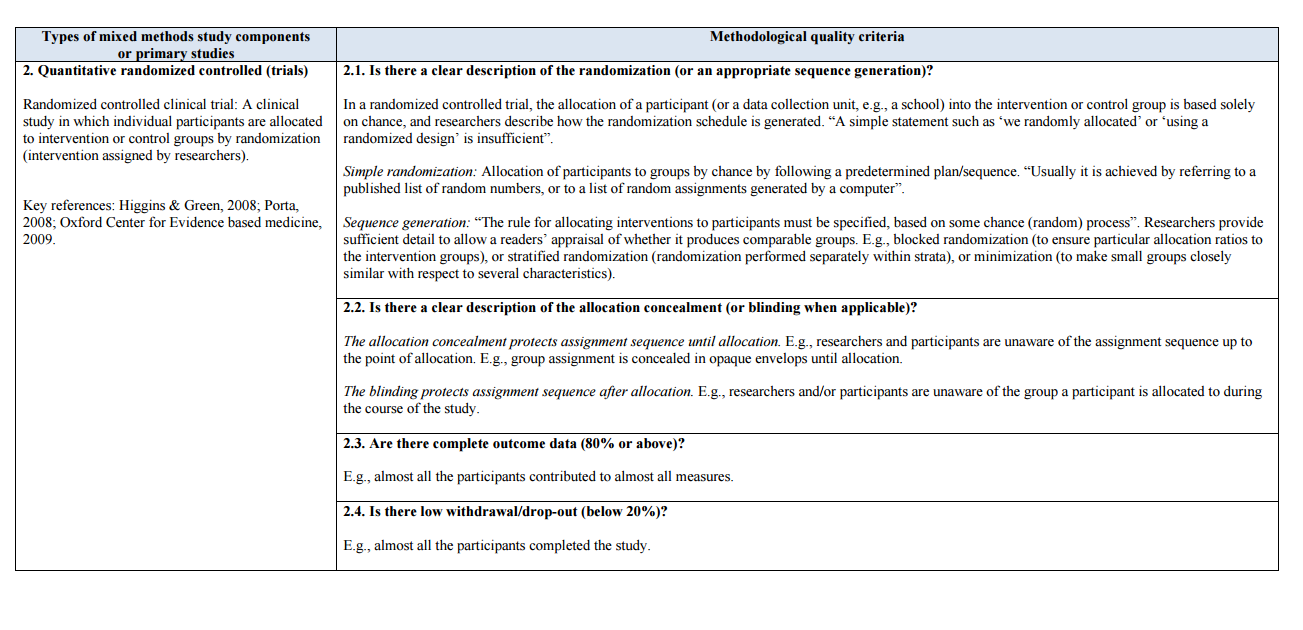


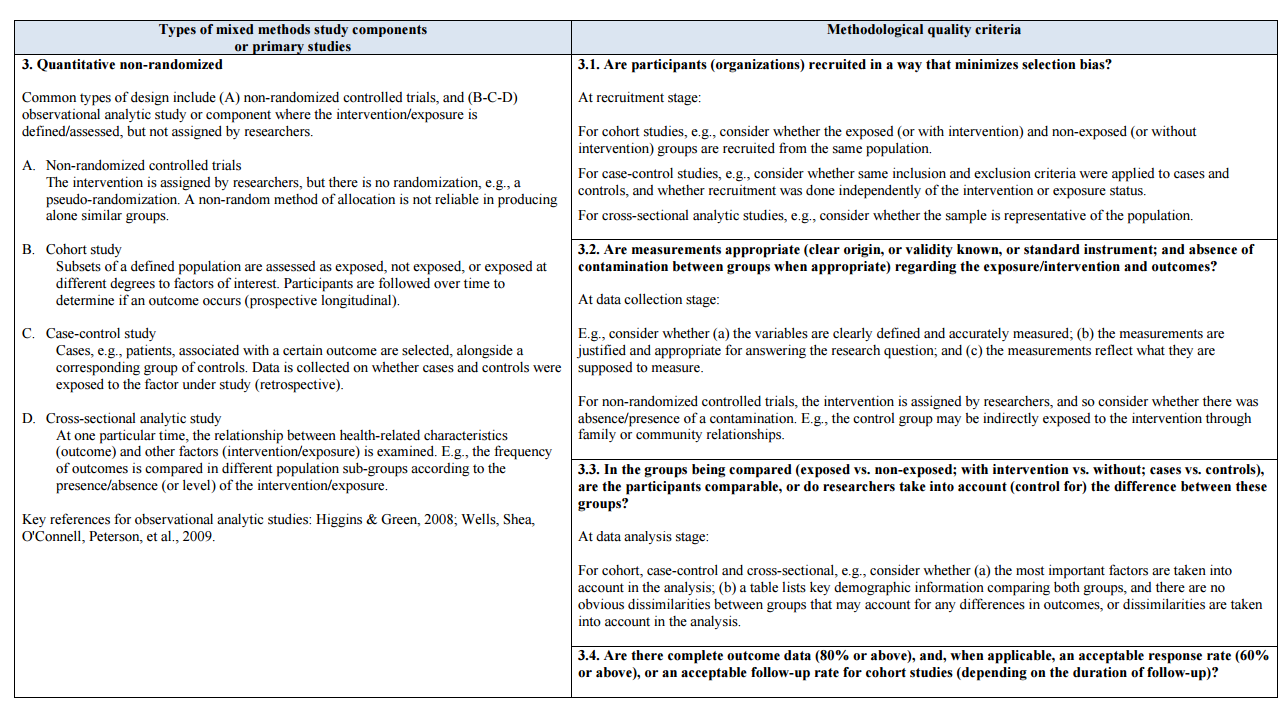


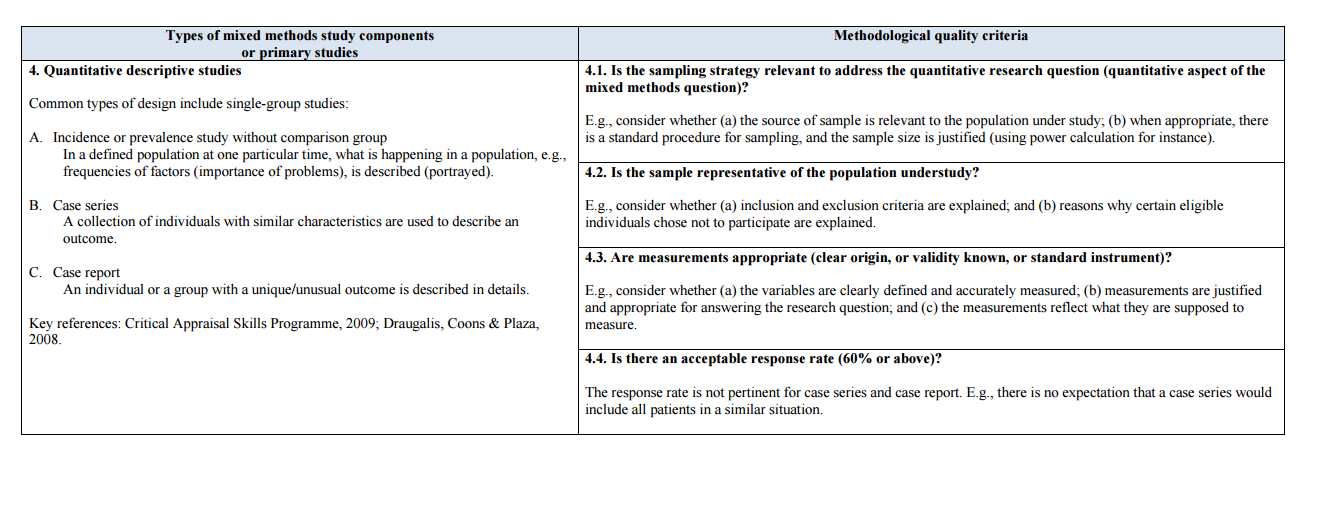


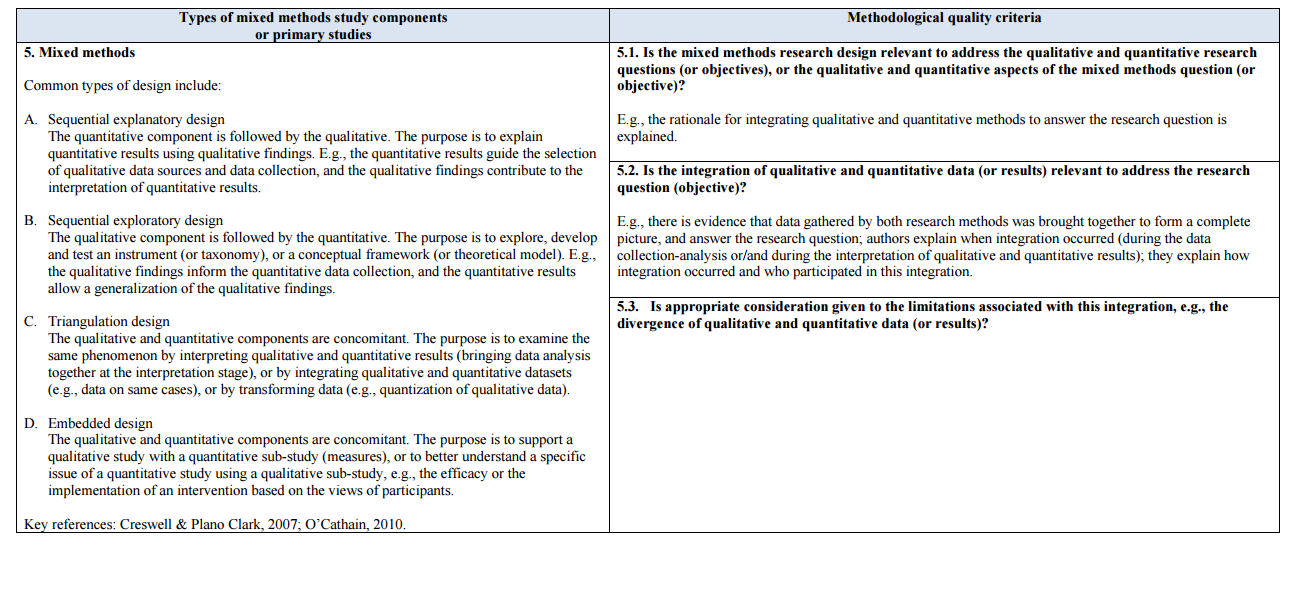

Supplement: Additional file: 2 — Study Selection, Data Collection and Quality Assessment Form. This is a pre-determined study eligibility form and data collection tool that will be used and consistently applied to reduce selection bias. (DOCX 1051 kb) [file 13643_2016_245_MOESM2_ESM.docx]
